# Medicinal Plants of *Solanum* Species: The Promising Sources of Phyto-Insecticidal Compounds

**DOI:** 10.1155/2022/4952221

**Published:** 2022-09-21

**Authors:** Kumarappan Chidambaram, Taha Alqahtani, Yahia Alghazwani, Afaf Aldahish, Sivakumar Annadurai, Kumar Venkatesan, Kavitha Dhandapani, Ellappan Thilagam, Krishnaraju Venkatesan, Premalatha Paulsamy, Rajalakshimi Vasudevan, Geetha Kandasamy

**Affiliations:** ^1^Department of Pharmacology and Toxicology, College of Pharmacy, King Khalid University, Al-Qara, Abha 61421, Saudi Arabia; ^2^Department of Pharmacognosy, College of Pharmacy, King Khalid University, Al-Qara, Abha, Saudi Arabia; ^3^Department of Pharmaceutical Chemistry, College of Pharmacy, King Khalid University, A-Qara, Abha, Saudi Arabia; ^4^Department of Biochemistry, Biotechnology and Bioinformatics, Avinashilingam Institute for Home Science and Higher Education for Women, Coimbatore 641043, Tamil Nadu, India; ^5^Department of Pharmacognosy, JKKMMRF's Annai JKK Sampoorani Ammal College of Pharmacy, Namakkal 638183, Tamilnadu, Tamil Nadu 638183, India; ^6^Faculty of Nursing, King Khalid University, Abha 61421, Saudi Arabia; ^7^Department of Clinical Pharmacy, College of Pharmacy, King Khalid University, Abha 61421, Saudi Arabia

## Abstract

Several medicinal plants have the potential to be a promising alternative pharmacological therapy for a variety of human illnesses. Many insects, including mosquitoes, are important vectors of deadly pathogens and parasites, which in the world's growing human and animal populations can cause serious epidemics and pandemics. Medicinal plants continue to provide a large library of phytochemicals, which can be used to replace chemically synthesized insecticides, and utilization of herbal product-based insecticides is one of the best and safest alternatives for mosquito control. Identifying new effective phyto-derived insecticides is important to counter increasing insect resistance to synthetic compounds and provide a safer environment. *Solanum* genus (Solanaceae family or nightshades) comprises more than 2500 species, which are widely used as food and traditional medicine. All research publications on insecticidal properties of Solanaceae plants and their phytoconstituents against mosquitoes and other insects published up to July 2020 were systematically analyzed through PubMed/MEDLINE, Scopus, EBSCO, Europe PMC, and Google Scholar databases, with focus on species containing active phytoconstituents that are biodegradable and environmentally safe. The current state of knowledge on larvicidal plants of *Solanum* species, type of extracts, target insect species, type of effects, name of inhibiting bioactive compounds, and their lethal doses (LC_50_ and LC_90_) were reviewed in this study. These studies provide valuable information about the activity of various species of *Solanum* and their phytochemical diversity, as well as a roadmap for optimizing select compounds for botanical repellents against a variety of vectors that cause debilitating and life-threatening human diseases.

## 1. Introduction

Medicinal plants are traditionally used to treat numerous human infections, and their bioactive compounds have long been important in therapeutic development, particularly in cancer and infectious diseases. Medicinal plant-derived natural products have garnered much interest in recent years as potential bioactive agents for insect vector control. Vector control is threatened by the emergence of resistance to conventional synthetic insecticides in vectors, among which mosquitoes pose high threats to human and animal health and life, often leading to the transmission of serious diseases, such as dengue, Ebola, filariasis, and malaria, resulting in millions of deaths each year [1–4]. Because chemical control of mosquitoes has been linked to such detrimental outcomes as the development of insect resistance, it is urgently necessary to discover and develop reliable and environmentally sustainable alternatives to current synthetic chemical insecticides.

As an alternative to synthetic insecticides, plant-based insecticide preparations have the advantages of rapid biodegradability and low toxicity to humans and animals [5]. Several plants and their constituents, especially those in medicinal herbs, have been traditionally used as insecticides, due to being rich in various bioactive phytochemicals and providing potential sources of natural mosquito control agents [6–9]. Recently, attention has been given to preparations of mosquito-larvicidal compounds based on herbal origin to enhance insecticidal effects and reduce the probability of development of resistance by the target pest population [10, 11]. While several plants from different families have been reported with mosquito-larvicidal properties, only a few species show promising effects and could be developed into natural insecticidal agents [12].

The *Solanum* family of plants is a large genus within the Solanaceae family that contains up to 2,000 species ranging from food crops to medicinal herbs. The genus *Solanum* has received much interest in chemical and biological studies over the last 30 years. Several steroidal saponins, steroidal alkaloids, disaccharides, flavonoids, and phenols have been implicated in the biological activities [13]. The genus *Solanum* appears to have a lot of potential, although most of the species are unknown or have had little research on their chemical contents. Several reviews of the *Solanum* genus and their phytochemistry have been published. These compounds have been linked to various health-promoting activities in the fight against several noncommunicable diseases, which are the leading causes of death worldwide. Many species belonging to this genus present a huge range of pharmacological activities such as anticancer, hepatoprotective, antimalarial, anthelmintic, and other activities [14]. Plants in this family are recognized for having a wide spectrum of alkaloid compounds, some of which are therapeutically the most potent. Steroidal glycoalkaloids are the most common and important group of nitrogen-containing secondary metabolites identified in Solanaceae plants. More than 350 *Solanum* species have yielded more than 100 different forms of glycoalkaloids [15, 16]. Many medicinal plants belonging to the Solanaceae family are promising therapeutic candidates to develop as bioinsecticidals against vector-borne human diseases such as malaria, leishmaniasis, and dengue fever due to the presence of different phytoconstituents. Various *Solanum* spp. provide a potential source of useful adulticidal drugs because of the presence of phytochemicals that can be used for the treatment of many diseases [17]. Thus, more scientific efforts should be made to identify and develop *Solanum*-based phyto-insecticides. Our literature review revealed 19 *Solanum* medicinal plants used in all parts (leaves, roots, bark, and flowers). The goal of this review is to compile most of the scientific literature on mosquito-larvicidal and insecticidal investigations of Solanum plants and their active bioactive chemicals from various scientific sources, including the types of extracts examined, dosages, and effect on target organisms.

## 2. Source of Data

A comprehensive systematic review of the literature up to July 2020 on Solanaceae plants with larvicidal effects present in standard electronic databases, such as EBSCO, Europe PMC, Google Scholar, MEDLINE, PubMed, Scopus, and Web of Science, was conducted using various keywords (adulticidal, botanical, essential oil, insecticidal, larvicidal, repellency, Solanaceae, *Solanum*, and steroidal alkaloids). The search was restricted to publications having English titles. In addition, a manual search was performed to categorize related articles using references from the retrieved literature.

A total of 51 full-text original research articles published in peer-reviewed journals on Solanaceae plants were retrieved, and data were culled for larvicidal effects. Roles of larvicidal activities were assessed in Solanaceae plant solvent extracts, such as acetone, chloroform, ethyl acetate, hexane, and methanol from seventeen different medicinal plants. Other parts of these plants with significant larvicidal properties against various mosquito vectors were highlighted.

## 3. Solanaceae Family

**Figure 1:**
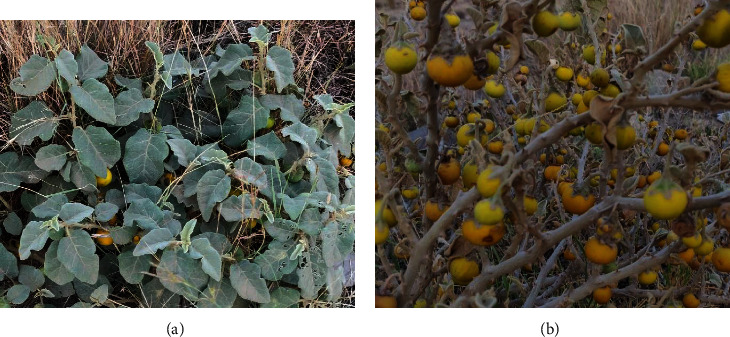
Leaves (a) and fruits (b) of *Solanum incanum*, Asir Region, Abha, Saudi Arabia.

*Solanum* L genus is the largest of the Solanaceae family or nightshades containing approximately 85–90 genera and 2,500–3000 species distributed in tropical and subtropical regions (Table 1) [12, 13]. Local names are given in various languages to describe a specific species for a particular local use. In Saudi Arabia, some species of Solanaceae are found primarily in the Asir Region and Jizan Region of Abha (Figure 1). A recent ethnobotanical study recorded three new collections of *Solanum* spp. in the southwest regions of Saudi Arabia [18].

### 3.1. Ethnopharmacological Use

Solanaceae is the most economically important family in the genus *Solanum* (Table 2). Solanaceae family offers a diversity of medicinal, culinary, and ornamental applications. The genus *Solanum* has attracted much interest in chemical and biological investigations over the last 30 years. Biologically important products for medicine and food include atropine, hyoscine, solasodine, and withanolide [19–21]. Although rich in alkaloids of medical importance, Solanaceae plants contain alkaloids with toxicity to humans and animals, ranging from mild irritation to fatal outcomes [22–25]. In addition, *Solanaceae* spp. have potential importance as food supplements worldwide [22, 26]. *S. nigrum, S. xanthocarpum*, *S. tuberosum,* and *S. lycopersicum* are a few economically important species of the *Solanum* genus. Various species in this genus have completed various pharmacological research to verify and validate their ethnopharmacological usage. However, various reviews of the *Solanum* genus have been published, most of which focused on a single species [14, 27–30].  Table 3 summarizes the scientific literature and reveals a variety of ethnopharmacologically based traditional insect repellents derived from *Solanum* plants utilized by local ethnic communities in various countries to avoid mosquito bites.

*Solanum* genus has several species found in tropical and subtropical areas and is used in folk medicine and dietary supplements. Among them, *S. nigrum* has been considered ethnobotanically important due to its use in the traditional healthcare system to cure various ailments. The leaves and bitter berries with pungent have been traditionally used against severe ulcers, heart diseases, piles, dysentery, gastritis, and stomachache [27]. *S. sisymbriifolium*, known as “wild tomato,” is a traditional medicine used by indigenous people of Central and South America to treat veterinary and human diseases. Various parts of the wild tomato have been widely used to prevent and treat numerous diseases, including hypertension, diarrhea, and respiratory and urinary tract infections [31].

*S. tuberosum* is used in folk medicine to treat burns, constipation, hemorrhoids, corns, cough, tumors, scurvy, and warts and to prevent wrinkles on the face [32]. *S. integrifolium* is native to Africa; its unripe fruits are eaten daily to check high blood pressure, inflammation, pain remedy to alleviate edema or cure stomach pain, lymphadenopathy, or sore armpits in indigenous medicine [34]. *S. villosum* is a traditionally important plant used in various systems of medicine for the treatment of leucorrhea, nappy rash, wounds, and cold sores, and as an ointment for sores and abscesses. A well-known traditional herb *S. xanthocarpum* is widely used in India to manage different ailments, including urolithiasis [35]. S. trilobatum is a widely used plant in the Indian indigenous systems of medicine. It is mainly used to treat respiratory diseases such as bronchial asthma [37]. *S. virginianum* L. has been used to manage fever, bronchial asthma, and cough for thousands of years [48].

In traditional medicine in Peru, *S. mammosum* is used to treat fungal infections and respiratory disorders via topical application. *S. incanum* is commonly found in Africa and is used as a folklore remedy for sore throat, angina, stomachache, colic, headache, wounds, pain relief in toothache, cure of snake bites, and sexually transmitted disease in wounds [49]. *S. elaeagnifolium* is called silverleaf nightshade and traditionally is used for the treatment of sore throats as an antiseptic agent, toothaches, and gastrointestinal disorders. Phytochemical analysis of berry extracts *S. elaeagnifolium* revealed the presence of kaempferol 8-C-*β*-galactoside that possesses medicinal proprieties [50]. *S. verbascifolium* is used in Chinese folklore for diarrhea, dysentery, eczema, edema, gout, headaches, ulcers, fever, hematuria, and toothache [45]. Despite being a poisonous plant, *S. pseudocapsicum* is used in traditional medicine to treat boils and gonorrhea and relieve abdominal pain, and as a male tonic [47]. *S. torvum* is another commonly used Solanaceae herb in traditional medicine. The plant extracts have been widely used to treat fever, wounds, tooth decay, reproductive problems, and arterial hypertension [46]. Thus, leaves, fruits, roots, and aerial parts of *Solanum* plants can benefit humans by enhancing their health when consumed as part of a daily diet, nutraceutical, or biopharmaceutical.

### 3.2. Phytopharmacology and Insecticidal Properties of Solanaceae spp

Medicinal Solanaceae plants have traditionally been used as insecticidal, anti-infectious, and antimicrobial agents [51, 52].  Table 4 shows the different types of test organisms, bioassays, and doses applied to investigate the mosquitocidal activity of crude plant extracts from the *Solanum* genus. Crude and chloroform-methanol extracts of *S*. *tuberosum* at very low concentrations are effective in mosquito control [54]. Volatile oils of *S*. *xanthocarpum* were effective as insect repellents, giving rise to >5 hours of protection against *Culex quinquefasciatus* without apparent dermal irritation to human skin [72]. Chloroform-methanol extract of *S*. *villosum* green berries was used as a biocontrol agent against *Aedes aegypti* [90]. *S. villosum* green berries had the greatest biocidal activity against *St. aegypti. aegypti*, and *Cx. quinquefasciatus* in chloroform and methanol extracts. As a result, crude extracts or protein fractions/isolated bioactive phytochemicals from *S. villosum* could be utilized as a possible biocontrol agent against these mosquitoes, especially because of its larvicidal impact [58, 59, 90]. *S. integrifolium* chitin-binding lectins (CBLs) inhibit *Spodoptera frugiperda* (sf21) insect cell growth by binding to carbohydrates and depolarizing mitochondrial membrane potential [60].

Table 5 summarizes detailed investigations of the mosquito-larvicidal efficiency of various *Solanum* species. Some examples are highlighted (according to the author's viewpoint) as follows: *S. xanthocarpum* extracts show various larvicidal and pupicidal activity against Cx's first-to-fourth instars*. Cx. quinquefasciatus* fruit aqueous extract exhibits 100% killing after 48-hour exposure compared with its root extract [61, 62]. The previous study has reported that the fruit extract of *S. xanthocarpum* and copepod *Mesocyclops thermocyclopoides* could serve as a potential highest mortality rate against dengue vector *Ae. aegypti* [63]. This mosquitocidal efficiency may be caused by detrimental effects of the *S. xanthocarpum* active principle compounds (solanocarpine and solanocarpidine) on the mosquito larvae. Similarly, *S. xanthocarpum* fruit extracts had larvicidal action against *An. stephensi* and *Cx. quinquefasciatus*, as well as one culicine species, *Ae. aegypti*. The toxic concentrations of fruit extract against *An. culicifacies*, *An. stephensi*, and *Ae. aegypti* were found to be 0.112 and 0.258%, 0.058 and 0.289%, and 0.052 and 0.218%, respectively, at the LC_50_ and LC_90_ levels. It was discovered that crude extracts have larvicidal capability due to their volatile oil content, implying that they could be used as an environmentally friendly, effective larvicidal in managing various vector-borne epidemics [66, 97]. Methanol leaf extract of *S*. *trilobatum* is effective against *Ae. aegypti*, *Cx. quinquefasciatus*, and *An. stephensi* pupae and larvae with an LC_50_ value of 125, 128, and 117 ppm, respectively [73]. Chloroform: methanol (1 : 1 v/v) extract of *S*. *nigrum* mature leaves is toxic against *Cx*'*s* early 3rd instar larvae of the *Cx. vishnui* group and *An. subpictus* [56].

The seed hexane extract of *S. trilobatum* exhibited (38%) acaricidal and insecticidal activities against the adult of *H. bispinosa* (Ixodidae) and hematophagous fly *H. maculata* Leach (Hippoboscidae). Therefore, this study provides the first report on the parasitic activities of plant extracts from southern India [75]. The leaf extract of *S. trilobatum* was found to have an oviposition deterrent effect, reducing egg-laying by *An. stephensi* by 18 to 99% and providing 70 to 120 minutes of mosquito bite protection skin repellent activities. *S. trilobatum* leaf extract had dose-dependent oviposition deterrent and skin repellent effects. Several solvent extracts of *S. trilobatum* were tested against the filarial vector *Cx. quinquefasciatus*; petroleum ether had the highest larvicidal activity, with LC_50_ values of 203.87 and 165.04, respectively, after 24 and 48 hours, followed by acetone and chloroform extracts [96]. According to the findings, *S. trilobatum* leaf extract is an efficient oviposition preventive and cutaneous repellent against *A. stephensi* [72]. The crude extract of the leaves or fruits of *S. incanum* and *W. somnifera* has an equal effect on the *A. messinae* mortality (96% mortality). However, the percentage mortality of the termite was 100% with 135 *µ*g from the crude extract of *S. incanum* leaves. Based on findings, both crude extracts have the potential to be used as termite control agents in termite breeding areas in the field or infested homes [80].

**Figure 2:**
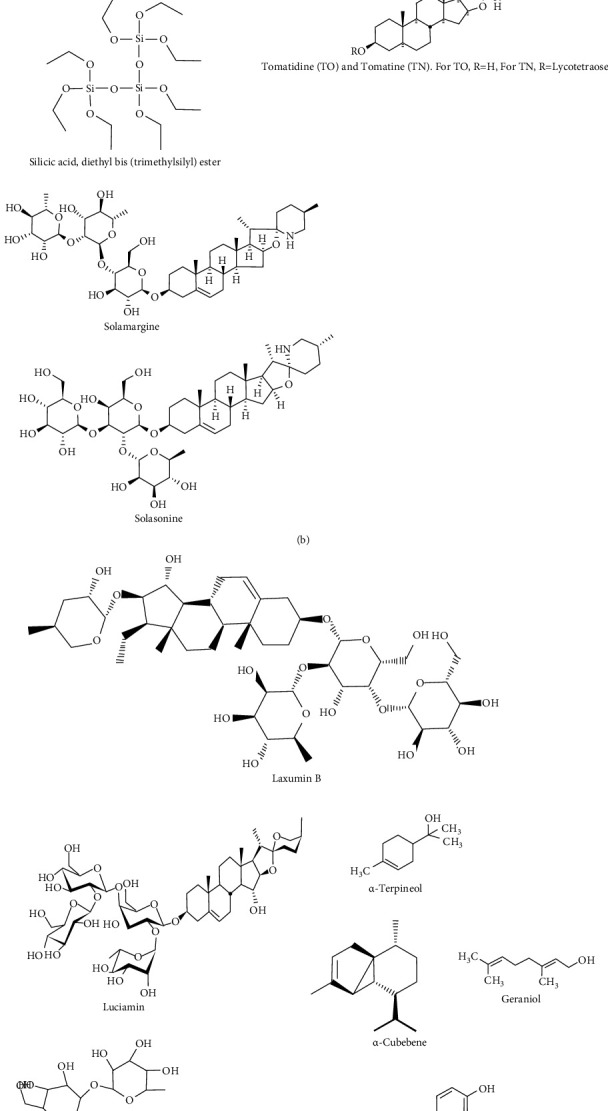
Chemical structures of insecticidal and mosquitocidal phytochemicals isolated from various *Solanum* species.

The larvicidal efficacy of *S. torvum* was tested against *An. stephensi* and *Cx. quinquefasciatus*, with the results indicating that the leaf methanol extract of *S. torvum* had the highest LC_90_, ranging from 70.38 to 210.68 ppm. As a result, isolated plant metabolites from *S. torvum* from southern India have the potential to be used as environmentally safe and long-lasting mosquito repellents [85]. The mosquito repellant effect of *S. lycopersicum esculentum* leaf hydro-ethanolic extract on the larvae of multiple mosquito species was tested at varied concentrations of 50, 100, 150, 200, and 250 ppm, with larva mortality seen within 24 hours. The hydro-ethanolic extract caused complete mortality in mosquitoes at 200 ppm in 18–19 hours, and the study found that *S. lycopersicum esculentum* may kill mosquitoes at a lower concentration [77]. The insecticidal effects of methanolic extracts of *S. elaeagnifolium* seeds were investigated further against *S. littoralis*, and 100% larval mortality was observed with the strongest growth inhibition (59.68%) compared to leaves [110]. Methanolic extracts from the leaves and seeds of *S. elaeagnifolium* also showed insecticidal efficacy against *P. operculella* and *T. castaneum*. Seed extract inhibited oviposition and egg hatching the most (95.9% and 98.6%, respectively), with an aphid mortality rate of 23.6% [79]. These findings suggested that numerous *Solanum* species might be used as plant-based mosquitocidal. They could be a valuable source for developing novel natural repellents as an alternative to chemical repellents in the future. The structure of phytochemicals with promising mosquitocidal and insecticidal effects from *Solanum* species is summarized in Figure 2.

### 3.3. Solanaceae spp. Phytochemicals with Insecticidal Properties

For decades, *Solanum* species have been widely used in healthcare systems as a source for various phytochemicals, including steroidal alkaloids. *Solanum* is distinguished by the presence of the steroidal alkaloid solasodine, which is a potential starting material for the manufacture of steroid hormones. Because of the wide spectrum of biological activities such as antibacterial, anti-inflammatory, antioxidant, and anticancer, *Solanum* alkaloids have been a topic of interest in pharmacological and therapeutical investigations. Because of metabolites such glycoalkaloids, some of the *Solanum* species are poisonous. Several pharmacologically important lead compounds are found in *Solanum* species, including steroidal alkaloids such as solasodine, solasonine, solamargine, and other medicinally important alkaloids; solasodine and its glycosylated derivatives, such as solamargine and solanine; and other chemicals with medicinal potential for developing new drugs against various human diseases.

*S. xanthocarpum* is an important source of many pharmacologically and medicinally useful alkaloids. Recent GC-MS analysis showed that several essential oils from leaves, fruits, roots, and stems of *S. xanthocarpum* were responsible for larvicidal activity [111]. Six important phytosteroids (1, 2-benzenedicarboxylic acid, dibutyl phthalate, phytol, lauric acid, 3,7,11,15-tetramethyl-2-hexadec, and 7-hexadecenal) with larvicidal activity against early 3rd instar larvae of the *Cx. vishnui* group were identified from mature leaves of *S. nigrum* using GC-MS [32]. A short polypeptide (15 amino acids) from mature leaves of *S. villosum* exhibited a moderate larvicidal effect. Further studies will be needed to determine its mode of action and appropriate formulations for field applications [90]. Eugenol and (E)-6-hydroxy-4,6-dimethyl-3-heptene-2-one in *S. nigrum* crude extract were proposed as being the main compounds responsible for mosquito-larvicidal activity [93]. The GC-MS analysis of acetone leaf extract of *S. trilobatum* revealed cyclodecanol (12.42%), *β*-sitosterol (10.25%) and 2-tetradecycloxirane (6.07%) as the major components and were possibly responsible for larvicidal activity against *Cx. quinquefasciatus* and *Ae. aegypti* [74].

*β*-Solamarine isolated from the methanolic extract of seeds of *S. elaeagnifolium* was found to have molluscicidal activity against *G. truncatula* and *F. hepatica*. The median lethal concentration of *β*-solamarine in molluscicidal activity (LC_50_) was of 0.49, and the study emphasizes that this glycoalkaloid may be used as molluscicides [101]. Another mosquitocidal investigation revealed that the leaf extract of *S. trilobatum* possesses oviposition deterrent and skin repellent activity against *An. stephensi*. Both oviposition deterrent and skin repellent activity were dose-dependent [112]. Two major compounds of steroidal glycoalkaloids were isolated from the fraction C MeOH extract of *S. sisymbriifoliu*m and were identified as solamargine (1) and *β*-solamarine. The toxicity of fraction C is 10-fold higher than that of *Anophelinae* larvae. These two steroidal alkaloids were known to possess molluscicidal activity where they could be used as a molluscicide in the future [53].

Luciamin, a spirostanol saponin, was isolated from the ethanolic extract of the aerial parts of *S. laxu*m and was tested against the aphid *S. graminum* by incorporation in artificial diets. Luciamin showed a deterrent (toxic) activity against the insect and is the first spirostanol glycoside reported to have this activity. Luciamin's aphid repelling effect deserves further exploration to determine its biological and economic effects against viral vectors [81]. Two *Solanum* glycosides isolated from *S. laxum* were found to have insecticidal effects against *S. graminum* with LC_50_ 4.3 *µ*M (laxumin A) and LC_50_ 6.1 *µ*M (laxumin B), respectively [82]. The insecticidal effect of the isolated steroidal alkaloids fraction B from *S. sisymbriifolium* was investigated on *Anophelinae* larvae (*A. gambia*, *A. funestus*). Compared with other extracts and fractions, fraction B, which contains solamargine and *β*-solamarine, appears less hazardous to larvae. As a result, fraction B could potentially be employed as an insecticide in the future [53]. *Solanum* species steroidal alkaloids are unique in their pharmacological properties and are important lead molecules for drug development [113].

Glucosisaustrin, a glucosinolate group of bioactive compounds from *S. nigrum*, was responsible for larval mortality. Glucosinolate is a plant-derived secondary metabolite and hydrophilic, having potent mosquito-larvicidal properties against *Cx. quinquefasciatus* and found to be safe for the environment [87]. For several decades, *N. glauca* has been known for its content of the pyridine alkaloids, such as anabasine, nicotine, and nornicotine. In the P*. rapae* larval bioassay, the median effective concentrations of anabasine were 0.572 mg/larva. Despite this, the insecticidal activities of the N. glauca extract are likely related to anabasine, as several phytochemical experiments and bioassays have shown [88]. This review intended to collect all of the scientific data on mosquitocidal, insecticidal, and larvicidal investigations on medicinally important *Solanum* compounds including steroidal alkaloids. Because most of the studies are laboratory-based and do not meet clinical standards, this comprehensive review is expected to bolster investigators in furthering their research into this field, which could lead to the development of plant-based mosquito repellents with significant economic benefits.

### 3.4. Solanum Plant-Mediated Nanoparticles

Plant extract-based silver nanoparticles have recently been developed to improve the control of mosquitoes without causing any significant harm to humans [114, 115]. According to the recent literature, silver nanoparticles (AgNPs) synthesized from aqueous extracts of *S. nigrum* and *S. mammosum* have demonstrated adulticidal and insecticidal activity against *Ae. aegypti*, implying that AgNPs produced from plant extracts have higher levels of toxicity than the extracts alone [55, 76]. Thus, these herbal-based AgNPs hold great promise as potent larvicides, but their environmental impact requires further investigation for controlling target vector mosquitoes.

## 4. Conclusion

Mosquito control is an important public health policy in tropical areas. Mosquitoes constitute a part of natural biodiversity, and their total eradication is not necessary, but only the diseases they transmit need to be eradicated. Nevertheless, bites from disease-bearing insects can be minimized and even avoided altogether. Inappropriate application of chemical insecticides in insect pest control can lead to insect resistance and pose environmental hazards and raises pest resistance to insecticides. Plants contain a variety of larvicidal secondary metabolites, and given their low environmental impact and minimal toxicity to humans, medicinal plants present a promising alternative to synthetic pesticides [116,117]. *Solanum* spp. constitute a large and diverse genus (∼2,000 species) of flowering plants, which provide food sources, eggplant, potato and tomato, ornamental flowers and fruits, and herbal medications. Solanum species grow in various habitats and can be annuals and perennials, vines, subshrubs, shrubs, and small trees.

*Solanum* spp. contain a diversity of phytochemicals that can be culled for their insecticidal properties, particularly mosquito larvicide, adulticide, and repellent. Although crude extracts have higher insecticidal potency than pure components, probably due to synergy among their bioactive constituents, optimal utilization of crude extracts is limited by an inability to control their contents, which can vary depending on plant species, cultivation conditions, the season of harvest, and extraction methods and solvents used. Thus, an understanding of the mechanism of insecticidal activity of the major bioactive compounds can lead to consistent and optimal formulation and adjustment to match the target insect pests of interest.

The review highlights current knowledge of phytocompounds (glycoalkaloids, phytosteroids, plant proteins, and volatile oils) reported as larvicides, adulticides, and repellents against a variety of insect pests, against vectors of important human diseases (dengue, filaria, and malaria). The exquisitely low larvicidal activity of silver nanoparticles formed from plant fruit and aqueous leaf extracts indicates the synergism between traditional medicinal herbal knowledge and modern (nano)technology. As a result, the usage of environmentally beneficial and cost-free plant-based products for insect/mosquito control is now unavoidable. Although the evaluation of phytochemicals is still in its early stages, with much more research needed to characterize promising agents and discover new ones, some of the findings presented in this review suggest that *Solanum* genus-based botanical phytochemicals should not be dismissed as a potential future alternative to synthetic insecticides. Hence, *Solanaceae* plants should be mined for their inexpensive, eco-friendly, safe, and effective alternatives to current chemical larvicides.

## Figures and Tables

**Table 1:** Taxonomy of *Solanaceae* family.

Taxonomic placement	Scientific division
Kingdom	Plantae
Subkingdom	Tracheobionta
Infrakingdom	Streptophyta
Superdivision	Spermatophyta
Division	Magnoliophyta/Tracheophyta
Class	Magnoliopsida
Subclass	Asteridae
Superorder	Asteranae
Order	Solanales
Family	Solanaceae
Subfamily	Nicotianoideae
Genus	Solanum L
Common name	Nightshade

**Table 2:** Common and scientific names of *Solanum* spp.

Common name	Scientific name
Silverleaf nightshade	*Solanum elaeagnifolium*
Scarlet eggplant	*S. integrifolium*
Sticky nightshade	*S. sisymbriifolium*
Potato	*S. tuberosum*
Black nightshade	*S. nigrum*
Red nightshade	*S. villosum*
Yellow-fruit nightshade	*S. xanthocarpum*
	*S*. *virginianum* L.
Thai nightshade	*S. trilobatum*
Indian ginseng (ashwagandha)	*Withania somnifera*
Nipplefruit nightshade	*S. mammosum*
Garden tomato	S. lycopersicum
Jasmine nightshade	*S*. *laxum*
Mullein nightshade	S. verbascifolium
Jerusalem cherry	S. pseudocapsicum
Thorn apple	S. incanum
Turkey berry	S. torvum

**Table 3:** List of various phytochemicals and ethnopharmacological uses from *Solanum* plants.

Species name	Medicinal uses	Parts used	Phytochemicals	Country used	References
*S. sisymbriifolium*	Used as contraceptive febrifuge, to treat syphilis, hypertension, diarrhea, and respiratory and urinary tract infections, and as analgesics	Whole plants	Solamargine and *β*-solamarine, cuscohygrine, sisymbrifolin, neolignan	Paraguay, India, Brazil, Perunad, and Argentina	[31]
*S. tuberosum*	Used to treat burns, constipation, hemorrhoids, corns, cough, tumors, scurvy, and warts, to prevent wrinkles on face, pain, acidity, and swollen gums, and to heal burns	Tubers, skins, raw juice	Solanidine, demissidine, *α*-chaconine, *α*-solanine, solavilline, solasdamine	Europe and South America	[32]
*S. nigrum*	Used to treat Liver disorders, diarrhea, inflammatory conditions, chronic skin ailments (psoriasis and ringworm), fever, hydrophobia, painful periods, and eye diseases	Whole plants	Steroidal alkaloids	Kenya, China, India, and Pakistan	[27]
			Steroidal saponins		
			Glycoprotein		
*S. villosum*	Used to treat leucorrhea, nappy rash, wounds, and cold sore	Whole plants	Solanidine, *α*-chaconine, (d) *α*-solanine	Africa, Central and South America, China, India, and Pakistan	[33]
*S. integrifolium*	Used to treat high blood pressure and edema or to cure stomach, lymphadenopathy, and inflammation, and as pain remedy to alleviate edema	Fruits	N-caffeoyl putrescine, 5-caffeoylquinic acid, and 3-acetyl-5-caffeoylquinic acid	South-East Asia, Brazil, Argentina, Uruguay, and Paraguay	[34]
*S. xanthocarpum*	Used to treat urolithiasis, respiratory disorders (expectorant, coughs, bronchial asthma, and chest pain), gonorrhea, pest repellent, tympanitis, misperistalsis, piles, and dysuria	Whole plants	Saponins, solanacarpine, solanacarpidine, solancarpine, solasonine	South-East Asia including India, Malaysia, and tropical Australia	[35, 36]
*S. trilobatum*	Used to treat cough and cold, respiratory disease (chronic bronchitis and tuberculosis), and male fertility, and to cure snake poison, dyspnea, anorexia, worm infestation, skin diseases, hemiplegia, edema, urinary calculi, amenorrhea, and urinary tract disorders.	Leaves	Sobatum, solasodine, solanine, tomatidine, diosgenin, soladunalinidine	China, Myanmar, Thailand, Vietnam, Sri Lanka, Peninsular Malaysia, and southern India	[37–39]
*S. virginianum*	Used to treat sore throats, chest pain and catarrh, stomach and respiratory complaints, fever, influenza, painful and difficult urination, bladder stones, and rheumatism	Whole plants	Arabinogalactan, chlorogenic and caffeic acid, khasianine, solasonine, solamargine, beta-solamargine, solanocarpine, and solanocarpidine	India, Sri Lanka, South-East Asia, Malaysia, tropical Australia, and Polynesia	[40]
*S. mammosum*	Used to treat fungal infections and respiratory disorders (asthma, cough, cold, and sinusitis), skin ulcer, scabies, furunculosis and rashes, insecticide, and rat poison	Leaves, fruits, and seeds	Indioside D, solamargine, tomatine, solasonine, diosgenin, solamargine and *β*-solamargine	Northern and South America, Caribbean islands, and Africa	[41]
S. lycopersicum	Used to treat skin and cardiovascular diseases, cancer, burns, scalds and sunburn, rheumatism and severe headaches, filarial worm swellings, incipient leprosy spots, and toothache.	Fruits	Lycopene, zeaxanthin, esculoside A, beta-carotene	South and Central America	[42]
*S. elaeagnifolium*	To cure cold and infant, typhoid, pneumonia, sore throats, an antiseptic agent, toothaches, and gastrointestinal disorders	Whole plants	Solanine, solasonine, solasodine, kaempferol 8-C-beta-galactoside, *β*-solamarine, solanidine	Asia, Africa, Australia, and tropical and subtropical America	[43]
*S. incanum*	Used to treat sore throat, angina, stomachache, ear inflammation, snake bites, wounds, liver disorders, skin ailments (ringworm), warts, inflammatory conditions, painful periods, and fever	Whole plants	Khasianine, incanumine, solasodine, kaempferol, isoquercitrin, yamogenin	Africa, Middle East and Far East Asia, and Arabian Peninsula	[44]
*S. jasminoides or S. laxum*	Used to kill insects	Aerial parts	Steroidal glycosides—inunigroside A; steroidal sapogenol—jasminoside A, solasodine, laxumin A, laxumin B	Uruguay, Brazil, South America, Paraguay, Uruguay, and Argentina	[13]
*S. pseudocapsicum*	Used to treat boils and gonorrhea, male tonic and abdominal pain, somnolence, and diabetes	Bark, fruit, leaves, and seeds	Solanocapsine, solacasine, solateinemine, O-methylsolanocapsine, episolacapine, and isosolacapine	India, Nepal, and the Philippines	[45]
*S*. *torvum*	Used to treat fever, wounds, tooth decay, reproductive problems, and arterial hypertension	Fruits and leaves	Chlorogenin, torvoside A-L, chlorogenone	Thailand, India, West Indies, and South America	[46]
*S. verbasicum*	Used to treat diarrhea, dysentery, eczema, edema, gout, headaches, ulcers, fever, hematuria, and toothache	Leaves and roots	Pentanone and *γ*-sitosterol	India and China	[47]

**Table 4:** Different types of test organisms, bioassays, and doses used to study the mosquitocidal activity of crude plant extracts from *Solanum* genus.

Species name	Species tested	Types of bioassays	Dose	References
*S. sisymbriifolium*	*Anophelinae* (insects and larvae)	Biocidal assay	0.005–5 g/ml	[53]
*S. tuberosum*	*Cx. quinquefasciatus* and *An. stephensi*	Larvicidal assay	1.1–0.5% (AE)	[54]
			1.2 25–75 ppm (CME)	
*S. nigrum*	*Cx. quinquefasciatus* and *An. stephensi*	Mosquito-larvicidal assay	2.5, 5, and 10 ppm	[55]
	*Cx. quinquefasciatus*	Larvicidal bioassay	1–3%	[56]
	*Cx. quinquefasciatus*	Mosquitocidal assay	15, 20, and 25 mg/L	[57]
*S. villosum*	*Cx. quinquefasciatus*	Larvicidal assay	30, 50, and 100 ppm	[58, 59]
	*An. subpictus*		30, 50, 100, and 200 ppm	
	S. aegypti		0.1–0.5% and 15, 25, and 30 ppm	
*S. integrifolium*	*Spodoptera frugiperda*	Insecticidal assay	1 *μ*g/mL	[60]
*S. xanthocarpum*	*Cx. quinquefasciatus*	Mosquito-larvicidal and pupicidal assays	50–650 ppm	[61]
	*Culicine larvae*	Larvicidal assay	1–5 ml	[62]
	*Ae. aegypti*	Mosquitocidal assay	100, 150, 200, 250, and 300 ppm	[63]
	*Ae. aegypti*	Insecticidal	0.82 mg/ml	[64]
	*Cx. quinquefasciatus*	Mosquito-larvicidal assay	62.5, 125, 250, 500, and 1000 mg/L	[65]
	*Cx. quinquefasciatus*	Larvicidal assay	7500–20 000 ppm	[66]
	An. stephensi	Larvicidal assay	1 : 1, 1 : 2, and 1 : 4%	[67–69]
	*Cx. quinquefasciatus*	Larvicidal assay	1 : 1, 1 : 2, and 1 : 4%	
	An. stephensi	Larvicidal assay	7500–20 000 ppm	[70]
	*Cx. Vishnui and L. acuminata*	Mosquito-larvicidal assay	75, 100, and 150 ppm	[71]
	*Cx. quinquefasciatus*	Mosquito-larvicidal assay	15, 20, and 25 ppm	
*S. trilobatum*	*An. stephensi*	Oviposition deterrent assay	0.01, 0.025, 0.05, 0.075, and 0.1%	[72]
		Skin repellent assay	0.001, 0.005, 0.01, 0.015, and 0.02%	[73]
	Ae. aegypti, Cx. quinquefasciatus, and An. stephensi	Larvicidal and pupicidal assays	50, 100, 150, 200, and 250 ppm	
	Ae. aegypti and Cx. quinquefasciatus	Larvicidal assay	100, 200, 300, 400, and 500 ppm	[74]
	*Haemaphysalis bispinosa* and *Hippobosca maculata*	Acaricidal and insecticidal assays	46.88 to 3,000 ppm	[75]
Solanum Mammosum-Silver Nanoparticles (Sm-AgNPs)	Ae. aegypti	Larvicidal assay	1500, 3000, 4500, and 6000 ppm	[76]
			0.05, 0.06, 0.07, and 0.08 ppm	
S. lycopersicum	Ae. aegypti and Cx. quinquefasciatus	Larvicidal activity	50, 100,150, 200, and 250 ppm	[77]
*S. elaeagnifolium*	*Fourth instar larvae*	Larvicidal assay	2% extract (5 *µ*l spray)	[78]
	*Tribolium castaneum* and *Phthorimaea operculella*	Insecticidal assay		[79]
*S. incanum*	*A. messinae and M. najdensis*	Insecticidal assay	2.5–135 *µ*g/ml	[80]
*S. laxum—laxumin A*	*Schizaphis graminum*	Insecticidal assay	50–500 *µ*m	[81]
*S. laxum—luciamin*	*Schizaphis* graminum	Repellant assay	50–500 *µ*m	[82]
*S. jasminoides*	*Phlebotomus papatasi and Bougainvillea glabra*	Insecticidal assay	—	[83]
*S. surattense*	*Callosobruchus chinensis*	Insecticidal assay	1, 2.5, 5, and 10%	[84]
*S*. *torvum*	*An. stephensi* and *Cx. quinquefasciatus*	Larvicidal bioassay	1.25 to 400 ppm	[85]
*S. verbasicum*	Cx. quinquefasciatus	Larvicidal activity	100, 300, 500, or 1000 ppm	[86]
*S. asperum*	*Biomphalaria glabrata*	Molluscicidal activity	10, 50, and 100 *µ*g/ml	[87]
*Nicotiana glauca*	*Pieris rapae*	Larvicidal assay	0.7 mg to 2.8 mg/ml	[88]
*S. elaeagnifolium*	*Tribolium castaneum*	Repellent and antifeedant assay	200 *μ*l/disc (2% extract)	[89]

**Table 5:** Insecticidal efficacy of Solanaceae plant extracts and their fraction/compound as adulticides.

*Solanum* spp.	Part used	Target insect species	Effect	Extract/compound fraction	Bioactive compound	LC_50_, LC_90_	Reference
*S. sisymbriifolium*	F	*Anopheles funestus and Anopheles gambiae*	Insecticidal	Total alkaloid fraction	Solamargine*β*-solamarine	0.45–0.75 mg/ml	[53]
*S. tuberosum*	T	*An. stephensi* (malaria vector)*Culex quinquefasciatus* (filaria vector)	Mosquito-larvicidal	Aqueous, chloroform: methanol (1 : 1)	NI	1.18–1.30 mg/l	[54]
*S. nigrum*	B, L	*An. stephensi* (malaria vector)*Cx. quinquefasciatus* (filaria vector)	Mosquito-larvicidal	Silver nanoparticle (AgNP)	Alkaloids	1.26–2.44 ppm	[55]
*S. villosum*	B	*Aedes aegypti* (dengue vector)	Mosquito-larvicidal	Chloroform:methanol	Steroids	21.02 (3^rd^ instar) ppm	[90]
*S. integrifolium*	F	*Spodoptera frugiperda*	Insecticidal	Chitin-binding lectins (CBLs) and crude	Polysaccharide	1 *µ*g/ml	[60]
*S. xanthocarpum*	L	*Cx. quinquefasciatus* (filaria vector)	Mosquito-larvicidal and pupicidal	Crude ethanolic	NI	155.3–448.4 ppm, 687.1–1,141.6 ppm	[61]
*S. xanthocarpum*	F	*Ae. aegypti* (dengue vector)	Mosquito-larvicidal and pupicidal	Crude ethanolic	SolanocarpidineSolanocarpine	253.2, 435.2 ppm79.5, 462.1 ppm	[63]
*S. xanthocarpum*	L	*Cx. quinquefasciatus* (filarial vector)	Mosquito repellent effect	Volatile oil	Volatile oil	8% repellency; 311 minutes of protection	[72]
*S. trilobatum*	L	*An. stephensi* (malaria vector)	Oviposition deterrent and skin repellent	Leaf extract	Volatile compounds	99.4% repellency; 123 minutes of protection	[72]
*S. xanthocarpum*	WP	*Cx. quinquefasciatus* (filaria vector)	Larvicidal and pupicidal	Chloroform fraction, crude	Quinine, terpenoids, and other compounds	227.9 ppm, 411.4 ppm	[91]
*S. xanthocarpum*	F, R	*Culicine larvae*	Larvicidal	Crude aqueous	AlkaloidsSaponins	∼100% mortality	[62]
*S. trilobatum*	L	*Ae. aegypti* (dengue vector)*An. stephensi* (malaria vector)*Cx. quinquefasciatus* (filaria vector)	Adulticidal	Crude methanolic	NI	116.6–127.8 ppm	[73]
*S. nigrum*	L	*An. subpictus Cx. vishnui* group	Larvicidal	Chloroform:methanol (1 : 1 v/v)	Phytosteroids	3.68–5.64 mg/l, 24.74–44.33 mg/l	[56]
*S. villosum*	L	*Ae. aegypti* (dengue vector)*An. stephensi* (malaria vector)*Cx. quinquefasciatus* (filaria vector)	Mosquito-larvicidal	Leaf protein	Polypeptides	644.7–747.2 ppm, 1,882.4–2,220.0 ppm	[90]
*S. virginianum*	WP	*Ae. aegypti* (dengue vector)	Insecticidal	Methanolic	NI	0.82 mg/ml	[92]
*S. nigrum*	B, L	*Ae. aegypti* (dengue vector)	Larvicidal	Crude	Eugenol (E)-6-hydroxy-4,6-dimethyl-3-heptene-2-one	Leaf: 9.8 ml/l, 26.4 ml/lGreen berry: 51.4 ml/l, 459.8 ml/lBlack berry: 9.9 ml/l, 56.1 ml/l	[93]
*S. nigrum*	L	*Cx. quinquefasciatus* (filaria vector)	Mosquito-larvicidal	Crude	AlkaloidsSteroids	0.08%, 0.37%	[57]
*S. nigrum*	B	*Cx. quinquefasciatus* (filaria vector)	Mosquito-larvicidal	Crude, chloroform: methanol (1 : 1, v/v)	Aromatic amide compounds	61.5 mg/l, 297.0 mg/l	[55]
*S. xanthocarpum, Withania somnifera*	F	*Ae. aegypti* (dengue vector)*An. stephensi* (malaria vector)*Cx. quinquefasciatus* (filaria vector)	Synergistic larvicidal	Crude aqueous	NI	SX: 48.4 mg/l, 218.2 mg/lSX : WS (1 : 1 v/v): 32.7 mg/l, 149.4 mg/lSX : WS (1 : 2 v/v): 22.9 mg/l, 109.8 mg/lSX : WS (1 : 3 v/v): 50.2 mg/l, 361.9 mg/l	[65]
*S. xanthocarpum*	WP	*Ae. aegypti* (dengue vector)*An. culicifaciesAn. stephensi* (malaria vector)*Cx. quinquefasciatus* (filaria vector)	Larvicidal	Methanolic	Edible oils	91.7–450.6 ppm, 379.0–1,881.0 ppm	[94]
*S. xanthocarpum*	NI	*Ae. aegypti* (dengue vector)*Cx. quinquefasciatus* (filaria vector)	Mosquito-larvicidal	Ethanolic	NI	788.10, 1288.91 mg/l,573.20, 1066.93 mg/l	[95]
*S. mammosum*	NI	*Ae. aegypti* (dengue vector)	Larvicidal	Aqueous silver nanoparticles	Steroidal alkaloids	1,631.3 ppm, 4,756.2 ppm; 0.06 ppm, 0.08 ppm	[76]
*S. trilobatum*	L	*Ae. aegypti* (dengue vector)*Cx. quinquefasciatus* (filaria vector)	Mosquito-larvicidal	Acetone	Cyclodecanol and other compounds	189.5 ppm, 444.3 ppm167.4 ppm, 371.8 ppm	[74]
*S. trilobatum*	AP	*Cx. quinquefasciatus* (filarial vector)	Mosquito-larvicidal	Acetone, chloroform, petroleum ether	NI	Acetone: 186.4 mg/l, 366.5 mg/lChloroform: 346.1 mg/l, 595.6 mg/lPetroleum ether: 165.0 mg/l, 293.5 mg/l	[96]
*S. xanthocarpum*	R	*An. stephensi* (malaria vector)	Larvicidal	Petroleum ether	NI	0.93 ppm, 8.48 ppm	[67]
*S. xanthocarpum*	R	*An. stephensi* (malaria vector)	Larvicidal	Petroleum ether with temephos (1 : 1)	NI	0.02 ppm, 0.09 ppm	[69]
*S. xanthocarpum*	F	*An. stephensi* (malaria vector)*Cx. quinquefasciatus* (filaria vector)	Larvicidal	Carbon tetrachloridepetroleum ether	NI	1.27 ppm, 59.45 ppm	[97]
*S. xanthocarpum*	R	*Cx. quinquefasciatus* (filaria vector)	Larvicidal	Petroleum ether	NI	38.48 ppm, 80.83 ppm	[66]
*S. xanthocarpum*	R	*Cx. quinquefasciatus* (filaria vector)	Larvicidal	Temephos:plant (1 : 1)	NI	0.01 ppm, 0.02 ppm	[67]
*S. xanthocarpum*	F, R	*Ae. aegypti* (dengue vector)*An. culicifaciesAn. stephensi* (malaria vector)	Larvicidal	Fruit, root	NI	0.05–1.16 ppm, 0.22–3.58 ppm	[98]
*S. villosum*	L	*Cx. quinquefasciatus* (filaria vector)	Larvicidal	Chloroform:methanol (1 : 1 v/v)	NI	39.19 ppm	[58]
*S. villosum*	L	*An. subpictus*	Larvicidal	Chloroform-methanol	Glycoalkaloids	23.47–30.63 ppm	[59]
S. lycopersicum	L	*Culex* and *Aedes* spp.	Larvicidal	Aqueous ethanolic	NI	100% mortality at 250 ppm	[77]
*S. nigrum*	L	*Ae. aegypti* (dengue vector)*An. culicifacies Cx. quinquefasciatus* (filaria vector)	Larvicidal	Crude aqueous	NI	0.027–0.032%, 0.027–0.212%	[99]
*S. elaeagnifolium*	F	*Blattella germanica*	Repellent effect	Ethanolic, hexane	NI	50 mg/ml	[100]
*S. elaeagnifolium*	L, B	*An. labranchiae*	Larvicidal effect	Aqueous, ethanolic	Glycoalkaloid extracts	LC_90_ (24 h) 209.8, 123.4 ppm	[78]
*S. elaeagnifolium*		*Fasciola hepaticaGalba truncatula Müll.*	Molluscicidal activity	Total saponin fractionTotal alkaloid fraction	*β*-solamarine	0.94 mg/L14.67 mg/L	[101]
*S. incanum*	F, L	*Amitermes messinaeMicrotermes najdensis*	Insecticidal	Crude hexane	*β*-chaconine, *α*-solanine	40% mortality at 67.5 *µ*g/ml	[80]
*S. jasminoides*	WP	*Phlebotomus papatasi* (Leishmania vector)	Larvicidal and repellent effect	Branch	NI	Median survival = 8 days (confidence interval: 17.1–18.9)	[83]
*S. nigrum*	F	*Ae. aegypti* (dengue vector)*An. culicifacies AAn. culicifacies CAn. stephensi* (malaria vector)*Cx. quinquefasciatus* (filaria vector)	Mosquito-larvicidal	Aqueous, hexane	NI	<20 ppm, <100 ppm	[102]
*S. pseudocapsicum*	NI	*Helicoverpa armigeraSpodoptera litura*	Antifeedant, insecticidal	Ethyl acetate (5%)	NI	Maximum insecticidal = 66.5–75.3%	[103]
*S. pseudocapsicum*	L, SD	*Agrotis ipsilon*	Antifeedant, insecticidal	Ethyl acetate (5%)	NI	Maximum insecticidal = 60.1%	[104]
*S. nigrum*	L, F	*Ae. caspiusCx. pipiens*	Larvicidal	70% ethanolic	NI	3.37 mg/l	[105, 106]
*S. surattense*	F, L, R, S	*Callosobruchus chinensis*	Pesticidal	Aqueous extract, aqueous suspension	NI	Reduction in oviposition = 2–5 eggs/pair	[84]
*S. surattense* and *S. trilobatum*	L	*Cx. quinquefasciatus* (filaria vector)	Insecticidal	Ethyl acetate, petroleum ether	NI	46.04 ppm	[107]
*S*. *torvum*	L	*An. stephensi* (malaria vector)*Cx. quinquefasciatus* (filaria vector)	Larvicidal	Methanolic	NI	(LC_90_) 70.38–210.68 ppm	[85]
*S. trilobatum*	L, SD	*Hippobosca maculata*	Insecticidal	Hexane	NI	495.61–432.77 ppm,1,914.84–1,872.33 ppm	[75]
*S. verbasicum*	L	*Cx. quinquefasciatus* (filaria vector)	Larvicidal	Various solvents	NI	100% mortality at 72 hours	[86]
*S. tuberosum*	T	*Leptinotarsa decemlineataEmpoasca fabae*	Insecticidal	Crude sample	ChaconineSolanine	100% defoliation	[108]
*S. laxum*	WP	*Schizaphis graminum*	Insecticidal	Steroidal glycoalkaloid fraction	Laxumin A and B	4.3 *μ*M and 6.1 *μ*M	[81]
*S. laxum*	AP	*Schizaphis graminum*	Insecticidal	Ethanolic	Luciamin	70% mortality at 24 hours	[82]
*S. nigrum*	B	*Cx. quinquefasciatus*	Mosquito-larvicidal	Chloroform: methanol (1 : 1, v/v) solvent	NI	80% mortality at 72 hours	[57]
*Solanum* steroidal alkaloids and glycoalkaloids	SA	*Tribolium castaneum*	Larvicidal	Isolated compounds in diet	Steroidal glycoalkaloids	89–100% larvicidal effect	[109]
*S. nigrum*	L	*Lymnaea acuminata Cx. vishnui(vector of Japanese encephalitis)*	Mosquito-larvicidal	Aqueous	Aliphatic amide compounds	LC_50_ 55.45 and 11.59 ppm, respectively at 72 h.	[70]
*S. nigrum*	L	*Cx. quinquefasciatus*	Mosquito-larvicidal	Ethyl acetate	Glucosisaustrin	32–48% mortality at 72 hours of 4th larval instars	[71]
*S. asperum*	L	*Biomphalaria glabrata*	Molluscicidal activity	Methanolic extractAlkaloidal fractionSolanandaineSolasonineSolamargine	SolanandaineSolasonineSolamargine	LC9044.1;17.3399.772.063.6	[87]
*Nicotiana glauca*	L	*Pieris rapae*	Larvicidal effect	Total alkaloid anabasine	Anabasine	EC50-1.202 mg/larva 0.572 mg/larva	[88]
*S. elaeagnifolium*	S, L	*Tribolium castaneum*	Insecticidal (repellent assay)	Methanol (seed and leaves)	Glycoalkaloids	% repellent effect:Seeds: 94% after 2 hrsLeaves: 74% after 2 hrs	[89]

AP: aerial part; B: berry; F: fruit; FL: flower; L: leaf; LC_50_: 50% lethal concentration; LC90: 90% lethal concentration; LC_100_: 100% lethal concentration; mg/l: milligrams per liter; mg/ml: milligrams per milliliter; ml/l milliliter per liter; *µ*g/ml: micrograms per milliliter; *μ*M: micromoles; NI: not identified; ppm: parts per million; R: root; S: stem; SA: *Solanum* alkaloids; SD: seed; SX: *S. xanthocarpum*; T: tuber; v/v: volume/volume; WP: whole plant; WS: *W. somnifera.*

## Data Availability

The data used to support the findings of the study can be obtained from the corresponding author upon request.
